# Relationship between amino acid ratios and decline in estimated glomerular filtration rate in diabetic and non-diabetic patients in South Africa

**DOI:** 10.4102/ajlm.v10i1.1398

**Published:** 2021-12-10

**Authors:** Thapelo Mbhele, Donald M. Tanyanyiwa, Refilwe J. Moepya, Sindeep Bhana, Maya M. Makatini

**Affiliations:** 1 Molecular Sciences Institute, School of Chemistry, Faculty of Science, University of the Witwatersrand, Johannesburg, South Africa; 2 School of Pathology, Faculty of Health Sciences, University of the Witwatersrand, Johannesburg, South Africa; 3 Department of Chemical Pathology, Faculty of Health Sciences, Sefako Makgatho Health Sciences University, Pretoria, South Africa; 4 School of Medicine, Faculty of Health Sciences, University of the Witwatersrand, Johannesburg, South Africa

**Keywords:** diabetic nephropathy, albuminuria, amino acids, LC-MS/MS, chronic kidney disease, glomerular filtration rate

## Abstract

**Background:**

Diabetic kidney disease is a major complication resulting from type 1 and type 2 diabetes. Currently, the microalbuminuria test is used to monitor renal function; however, it does not detect albumin until progressive loss of renal function has occurred.

**Objective:**

This study analysed the relationship between changes in amino acid ratios and estimated glomerular filtration rate (eGFR) decline in diabetic and non-diabetic patients.

**Methods:**

Urine samples were collected from participants between February 2019 to April 2019 and analysed from November 2020 to January 2021. Diabetic (glycated haemoglobin > 6.4%) and non-diabetic patients (glycated haemoglobin ≤ 6.4%) from Chris Hani Baragwanath Hospital, South Africa, were further categorised based on the degree of renal function predicted by the eGFRs. Amino acids were quantified using tandem mass spectrometry to determine the concentrations and ratios of tyrosine/phenylalanine, ornithine/arginine, arginine/citrulline and citrulline/ornithine at different stages of the chronic kidney disease.

**Results:**

Among diabetic patients, the tyrosine/phenylalanine ratio showed a statistically significant increase (*p* = 0.04) as the eGFR declined from stage 1 to stage 4; the ornithine/arginine ratio showed a strong negative correlation with eGFR. The citrulline/ornithine ratio differed between the diabetic and non-diabetic patients in stage 1 of chronic kidney disease.

**Conclusion:**

Amino acid ratios (ornithine/arginine and tyrosine/phenylalanine) are affected by the progression of diabetes and can be correlated to renal function. The citrulline/ornithine ratios differ between the studied groups in stage 1 of the disease and may be utilised to predict the onset of chronic kidney disease.

## Introduction

Diabetic nephropathy or chronic kidney disease (CKD), resulting from both type 1 and type 2 diabetes, is one of the leading causes of death in the world among non-communicable diseases.^1,2^ Diabetic nephropathy usually develops about 10 years after the onset of diabetes.^3^ Chronic kidney disease affects about half a million people globally, and people with CKD are at a high risk of developing stroke and cardiovascular disease. Chronic kidney disease patients are most likely to die from cardiovascular disease than progress to end-stage renal failure.^4^

In South Africa, diabetic nephropathy is diagnosed clinically using a digital community analyser vantage instrument that measures the amount of albumin and creatinine, as well as the ratio of albumin to creatinine (A/C) in urine. The diagnostic criteria based on the A/C ratio are as follows: normal range ≤ 3.5 mg/mmol (female) and ≤ 2.5 mg/mmol (male); microalbumin range 2.6 mg/mmol – 25.0 mg/mmol (female) and 3.6 mg/mmol – 35.0 mg/mmol (male); macroalbumin range 26.0 mg/mmol – 100.0 mg/mmol (female) and 36.0 mg/mmol – 100.0 mg/mmol (male).^5^ Although the A/C ratio is a good indicator of CKD, the detected value needs to be sustained in the patient over 3 months to accurately diagnose CKD.^6^ It also lacks specificity and sensitivity during a progressive decline in renal function and can give false results, where patients with CKD could appear normal.^7^

The glomerular filtration rate (GFR) is an important marker in the assessment of kidney function. It can be calculated using the CKD Epidemiology Collaboration formula and the modification of diet in renal disease equation. The modification of diet in renal disease equation estimates the GFR (eGFR) based on age, sex and serum creatinine (SCr).^7,8^ Renal decline in CKD is classified into five stages, which indicate the progression of the disease. These stages are based on the GFR and include stage 1 (≥ 90 mL/min per 1.73 m^2^), stage 2 (60 mL/min – 89 mL/min per 1.73 m^2^), stage 3 (30 mL/min – 59 mL/min per 1.73 m^2^), stage 4 (15 mL/min – 29 mL/min per 1.73 m^2^) and stage 5 (< 15 mL/min per 1.73 m^2^).^9^

The assessment of GFR using SCr concentrations gives inconclusive results in the early stages of renal decline and lacks sensitivity because SCr only becomes detectable after renal decline has progressed significantly. The CKD Epidemiology Collaboration formula provides a good indicator of the eGFR in the early stages of CKD (> 90 mL/min per 1.73 m^2^); however, it also underestimates the GFR in diabetic patients.^10^ Similarly, although the modification of diet in renal disease equation provides reliable results for patients with CKD, it may give false positives for healthy patients.^8^

Measuring the concentration of amino acids in the body is important in the diagnosis and management of several metabolic abnormalities. Amino acid concentrations can be used to determine the status of patients’ nutrition, renal function and tissue injury.^11^ The metabolism and excretion of amino acids such as phenylalanine and tyrosine have been reported to undergo considerable changes in patients with CKD.^12^ Urine is the most preferred biofluid in metabolomics due to its ease of collection in large volumes and its lesser complexity compared to other body fluids such as blood.^13,14^

Liquid chromatography tandem mass spectrometry eliminates lengthy sample preparations as amino acids can be analysed without the need for derivatisation.^11^ The advantages of using this technique include high sensitivity, specificity, and throughput.^15^ This technique is based on the structural characteristics of analytes obtained by fragmentation through collision, and it is a powerful technique for quantitation.^16^

This study aimed to assess the relationship between urinary amino acid concentration ratios and renal function decline as predicted by the eGFR in diabetic and non-diabetic patients using liquid chromatography tandem mass spectrometry.

## Methods

### Ethical considerations

Ethical clearance was obtained from the Human and Animal Rights Chris Hani Baragwanath Academic Hospital Medical Advisory Committee and the University of the Witwatersrand Human Research Ethics Committee (reference number: M180798). A brief explanation of the study was communicated to the participants, who then provided written informed consent to allow the collection and analysis of their urine samples and medical records. Medical practice and ethical guidelines were followed to ensure the confidentiality of the results obtained in this study. Samples were coded to preserve anonymity.

### Study design and sampling

A total of 187 spot urine specimens were collected from study participants between February 2019 and April 2019 at the Chris Hani Baragwanath Academic Hospital located in Soweto, Johannesburg, South Africa. This is the biggest hospital in Africa and serves a large community from urban to rural areas; it sees hundreds to thousands of patients annually. Data on ethnicity, age, eGFR, glycated haemoglobin (HbA1c), SCr, urinary creatinine, albumin, A/C and gender were obtained from participants’ medical records.

Samples from 36 participants were excluded from the analysis because of missing data (33 medical records without eGFR data and 3 without HbA1c data). All participants with complete medical data (*n* = 151) were of African descent, including women (*n* = 104) and men (*n* = 47) between the ages of 18 years and 90 years.

Urine samples were divided into two groups based on HbA1c: people with diabetes (*n* = 129; HbA1c > 6.4%) and people without diabetes (*n* = 22; HbA1c ≤ 6.4%). These groups were further divided into sub-groups based on the stage of CKD as follows: 63 people with diabetes and 9 without diabetes were categorised as being in stage 1 (≥ 90 mL/min per 1.73 m^2^), 43 people with diabetes and 10 without diabetes were classified as being in stage 2 (60 mL/min – 89 mL/min per 1.73 m^2^), 15 people with diabetes and 3 without diabetes were classified as being in stage 3 (30 mL/min – 59 mL/min per 1.73 m^2^), 7 people with diabetes were classified as being in stage 4 (15 mL/min – 29 mL/min per 1.73 m^2^), and 1 participant was categorised as being in stage 5 (< 15 mL/min per 1.73 m^2^).

The amino acids arginine, citrulline, isoleucine, ornithine, phenylalanine, and tyrosine were quantitatively analysed in each sample. Using the modification of diet in renal disease equation, the eGFR was calculated to assess the degree of kidney function. Urine samples were collected into 20 mL sterile polypropylene screw-top containers and placed on wet ice in polystyrene containers before transportation to the National Institute of Occupational Health biobank for long-term storage at −20 °C. Before analysis, the samples were transported to the University of the Witwatersrand School of Chemistry mass spectrometry laboratory, where they were briefly stored at −20 °C for a few days before analysis.

### Chemicals and materials

All amino acids (purity ≥ 98.0%), as well as the formic acid (purity ≥ 98.0%), Sigmatrix Urine Diluent (purity ≥ 98.0%) and HPLC-grade acetonitrile (≥ 99.9%), were obtained from Sigma-Aldrich (Johannesburg, South Africa). Deionised water, which was used as a solvent for dilution of analytes, was purified using a Direct-Q^®^ 3 UV Millipore system (Molsheim, France).

A stock solution of each amino acid (100 mg/L) was prepared by weighing 10 mg of the amino acid which was then dissolved to a final volume with Millipore H_2_O in a 100 mL volumetric flask. From the stock solution, 10 calibration points (0.01 mg/L, 0.05 mg/L, 0.10 mg/L, 0.30 mg/L, 0.50 mg/L, 1.00 mg/L, 2.00 mg/L, 3.00 mg/L, 4.00 mg/L, and 5.00 mg/L) were prepared by diluting appropriate volumes of stock solution in a urine diluent–H_2_O (1:5 ratio) mixture. The urine diluent was used as a blank matrix to mimic human urine.

### Quantitative analysis

Frozen urine samples were thawed for 30 min, then 1 mL of the urine was transferred into a 2 mL centrifuge tube and diluted with 1 mL of Millipore H_2_O, and centrifuged at 3000 g for 10 min. Afterwards, 0.2 mL of the supernatant was transferred into a 2 mL ultra-high performance liquid chromatography (UHPLC) vial and 0.8 mL of Millipore H_2_O was added to make up the final volume to 1 mL. Standards and samples were analysed on a YMC-Triart C18 UHPLC column (1.9 µm beads, pore size 120 Å, 2 mm ID *×* 150 mm length) (Analytical Science Technology Pty Ltd, Cape Town, South Africa). All analyses were conducted in triplicate with quality control samples (lowest limit of quantitation [LLOQ]1 [0.1 mg/L], LLOQ3 [0.5 mg/L] and upper limit of quantitation [ULOQ] [4 mg/L]), which were analysed in the same way as the urine samples above and were used to assess the performance (linearity, accuracy, precision, and percentage recovery) of the method.

The Thermo Scientific^TM^ UltiMate^TM^ 3000 UHPLC system coupled to a Bruker Compact Qq-Time-of-Flight high-resolution mass spectrometer (Bruker Daltonics, Bremen, Germany) was used for the analysis. The samples and standards were placed in an autosampler set at 5 °C. A two-buffer system was employed, utilising formic acid as the ion-pairing agent; solvent A consisted of 0.1% formic acid in H_2_O (volume per volume), and solvent B comprised 0.1% formic acid in acetonitrile (volume per volume). Twenty µL of the sample was injected into the C18 UHPLC column and eluted using a gradient elution technique, starting from an H_2_O:acetonitrile ratio of 95:5% to 5:95% in 10 min; the standards and samples were injected three times.

#### Mass spectrometry parameters

The electrospray ionisation source was operated in the positive ionisation mode at a temperature of 250 °C. The dry gas (N_2_) flow rate was set at 9.0 L/min, the nebuliser gas (N_2_) pressure was set to 1.8 bars, the capillary voltage was 4500 V, the end plate offset was -500 V, and the charging voltage was 2000 V.

In the quadrupole, a multiple reaction monitoring method was applied with a mass range of 50 mass-to-charge ratio (m/z) to 1300 m/z to fragment arginine (175 m/z), citrulline (176 m/z), isoleucine (132 m/z), ornithine (133 m/z), phenylalanine (166 m/z), and tyrosine (182 m/z) at 26.00, 14.00, 22.00, 12.00, 15.00 and 12.00 eV collision energies. The time-of-flight detector voltage was set at 2242.9 V.

### Data analysis

All data processing was done on the Bruker QuantAnalysis software (Bruker Daltonics, Bremen, Germany) to determine the concentration of amino acids. The dilution factor of 10 was used to determine the actual concentration of amino acids in the urine samples. The LLOQ was determined for each amino acid and found to be within the range of 0.52 mg/L to 0.58 mg/L for arginine, citrulline, and ornithine and 0.12 mg/L to 0.19 mg/L for isoleucine, phenylalanine, and tyrosine (Table 1). Statistical data analysis was carried out in Microsoft Excel version 2101 (Microsoft Corporation, Redmond, Washington, United States). Mean and standard deviation values were obtained using descriptive statistics, significant differences in variables between diabetic and non-diabetic patients were assessed using analysis of variance, and linear correlations between biochemical and analytical data were determined using Pearson’s correlation coefficient (*R*). A *p*-value 0.05 or less was considered significant.

**TABLE 1:** Analytical method performance characteristics of three spiked blank matrices with amino acid standards at different concentrations, Johannesburg, South Africa, November 2019 to January 2021.

Target analyte	Linearity *R*^2^	Concentration range (mg/L)	Retention time (min)	Lowest limit of detection (mg/L)	Lowest limit of quantification (mg/L)	% Recovery ± % relative standard deviation		
						Blank matrix spiked with (0.1 mg/L) amino acid	Blank matrix spiked with (0.5 mg/L) amino acid	Blank matrix spiked with (4 mg/L) amino acid
Arginine	0.987	0.01–5	0.81	0.17	0.52	111.16 ± 26.68	113.10 ± 16.58	101.02 ± 9.70
Citrulline	0.983	0.01–5	0.90	0.19	0.58	155.99 ± 13.03	103.66 ± 8.08	107.97 ± 12.78
Isoleucine	0.999	0.01–5	2.10	0.05	0.16	108.72 ± 19.05	110.21 ± 10.75	107.08 ± 5.00
Ornithine	0.985	0.01–5	0.74	0.18	0.55	118.96 ± 50.56	124.42 ± 18.09	108.89 ± 17.72
Phenylalanine	0.999	0.01–5	4.44	0.04	0.12	99.08 ± 8.95	125.29 ± 2.80	120.92 ± 1.45
Tyrosine	0.998	0.01–5	2.96	0.06	0.19	90.54 ± 6.45	130.53 ± 2.69	120.92 ± 1.15

Note: Linear (Pearson’s correlation) regression was used to determine *R*^2^.

## Results

**FIGURE 1:**
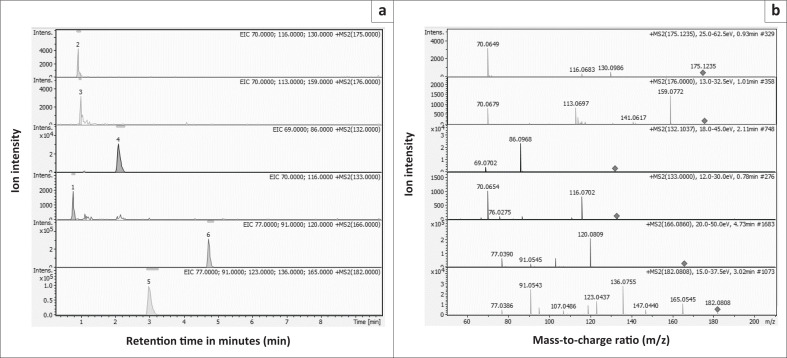
Chromatogram showing (a) the UHPLC baseline separation of arginine, citrulline, isoleucine, ornithine, phenylalanine and tyrosine; and (b) the mass spectrum with retention times and collision energy required to fragment each amino acid to give the fragment ions (quant masses). Data analysed between November 2019 and January 2021, Johannesburg, South Africa.

All amino acids were well separated on the C18 column and gave different fragment ions and retention times (arginine, 0.81 min; citrulline, 0.90 min; isoleucine, 2.10 min; ornithine, 0.74 min; phenylalanine, 4.44 min and tyrosine, 2.96 min), indicating good selectivity (Figure 1).

In diabetic patients, the ornithine/arginine and tyrosine/phenylalanine ratios increased as the eGFR decreased (Table 2). This relationship was confirmed by strong negative correlations (*R* = –0.81; *p* = 0.19 and *R* = –0.96; *p* = 0.04) between the amino acid concentration ratios and the eGFR. Conversely, the arginine/citrulline ratio decreased with decreasing eGFR, and this was supported by a statistically significant positive correlation (*R* = 0.71; *p* = 0.29). The concentrations of ornithine, isoleucine, tyrosine and phenylalanine decreased with decreasing renal function and this was confirmed by statistically significant correlations ([*R* = 0.83; *p* = 0.17], [*R* = 0.94; *p* = 0.06], [*R* = 0.94; *p* = 0.06] and [*R* = 1.00; *p* = 0.004]). The amino acid concentrations demonstrated statistically significant positive correlations to HbA1c coefficient (R) and correlated negatively with urinary creatinine and age. There was no significant correlation between the amino acid concentrations and A/C ratio, albumin, or SCr. Haemoglobin A1c correlated strongly with concentrations of citrulline (*R* = 0.76), isoleucine (*R* = –0.83) and ornithine (*R* = –0.86), while age correlated negatively with the concentrations of isoleucine (*R* = –0.98), ornithine (*R* = –0.95), phenylalanine (*R* = –0.83) and tyrosine (*R* = –0.80).

**TABLE 2:** Correlation between the amino acid concentrations and ratios and clinical data of patients with diabetes, Johannesburg, South Africa, November 2019 to January 2021.

Target analyte	HbA1c (%)		A/C (mg/mmol)		Albumin (mg/L)		UCr (mmol/L)		SCr (mg/dL)		Age (years)		eGFR (mL/min per 1.73 m^2^)	
	*R*	*p*	*R*	*p*	*R*	*p*	*R*	*p*	*R*	*p*	*R*	*p*	*R*	*p*
Arg	0.60	0.40	0.16	0.84	−0.68	0.32	−0.88	0.12	−0.07	0.93	−0.67	0.33	0.56	0.45
Cit	0.76	0.24	0.34	0.66	−0.28	0.72	−0.94	0.06	0.16	0.84	−0.65	0.35	0.33	0.67
Ile	0.83	0.17	−0.53	0.47	−0.39	0.61	−0.84	0.16	−0.69	0.31	−0.98	0.02	0.94	0.06
Orn	0.86	0.14	−0.28	0.72	−0.44	0.56	−0.95	0.05	−0.46	0.54	−0.95	0.05	0.83	0.17
Phe	0.54	0.46	−0.75	0.25	−0.53	0.47	−0.55	0.45	−0.88	0.12	−0.83	0.17	1.00	0.004
Tyr	0.53	0.47	−0.48	0.52	−0.74	0.26	−0.68	0.32	−0.67	0.33	−0.80	0.20	0.94	0.06
Cit/Orn	0.46	0.54	0.75	0.25	0.06	0.94	−0.61	0.39	0.66	0.34	−0.17	0.83	−0.25	0.75
Arg/Cit	−0.04	0.96	−0.90	0.10	−0.37	0.63	0.12	0.88	−0.92	0.08	−0.32	0.68	0.71	0.29
Orn/Arg	−0.12	0.88	0.48	0.52	0.88	0.12	0.32	0.68	0.66	0.34	0.49	0.51	−0.81	0.19
Tyr/Phe	−0.40	0.60	0.67	0.33	0.69	0.31	0.49	0.51	0.83	0.17	0.73	0.27	−0.96	0.04

Note: Pearson’s correlation was used to calculate *R* and *p*-values. *p* > 0.05, not significant; *p* ≤ 0.05, significant.

A/C, albumin creatinine ratio; Arg, arginine; Cit, citrulline; eGFR, estimated glomerular filtration rate; HbA1c, haemoglobin A1c; Ile, isoleucine; Orn, ornithine; Phe, phenylalanine; *R*, correlation coefficient; SCr, serum creatinine; Tyr, tyrosine; UCr, urinary creatinine.

The trends observed between amino acid concentrations and ratios with clinical parameters for non-diabetic patients are within the normal range for HbA1c and eGFR, and hence do not show disease progression (Table 3). Serum creatinine correlated significantly (*p* = 0.45) with isoleucine concentration (*R* = 0.76) and citrulline/ornithine ratios (*R* = 0.81, *p =* 0.40) in non-diabetic patients. The tyrosine/phenylalanine ratios (*R* = 0.85), as well as the concentrations of tyrosine (*R* = 0.76) and ornithine (*R* = 0.83), decreased with decreasing eGFR, while the concentrations of citrulline (*R* = –0.94) and isoleucine (*R* = –0.91) increased with decreasing eGFR (Table 3).

**TABLE 3:** Correlation between the amino acid concentrations and ratios and clinical data of non-diabetic patients, Johannesburg, South Africa, November 2019 to January 2021.

Target analyte	HbA1c (%)		A/C (mg/mmol)		ALB (mg/L)		UCr (mmol/L)		SCr (mg/dL)		Age (years)		eGFR (mL/min per 1.73 m^2^)	
	*R*	*p*	*R*	*p*	*R*	*p*	*R*	*p*	*R*	*p*	*R*	*p*	*R*	*p*
Arg	0.69	0.52	−0.31	0.80	−0.09	0.94	0.00	1.00	−0.16	0.90	0.48	0.68	−0.13	0.92
Cit	0.56	0.62	−0.87	0.33	−0.96	0.19	−0.98	0.13	1.00	0.04	0.76	0.45	−0.94	0.22
Ile	0.98	0.14	−0.97	0.15	−0.90	0.29	−0.85	0.35	0.76	0.45	1.00	0.03	−0.91	0.27
Orn	−0.34	0.78	0.71	0.49	0.85	0.35	0.89	0.30	−0.95	0.20	−0.57	0.61	0.83	0.38
Phe	−0.22	0.86	−0.23	0.86	−0.44	0.71	−0.52	0.66	0.64	0.56	0.04	0.97	−0.40	0.74
Tyr	−0.22	0.86	0.62	0.57	0.78	0.43	0.83	0.37	−0.91	0.27	−0.47	0.69	0.76	0.46
Cit/Orn	0.02	0.99	−0.45	0.70	−0.64	0.56	−0.71	0.50	0.81	0.40	0.28	0.82	−0.61	0.58
Arg/Cit	0.06	0.96	0.38	0.75	0.57	0.61	0.64	0.55	−0.75	0.46	−0.20	0.87	0.54	0.64
Orn/Arg	−0.20	0.87	−0.25	0.84	−0.46	0.70	−0.53	0.64	0.66	0.54	0.06	096	−0.42	0.73
Tyr/Phe	−0.38	0.76	0.74	0.47	0.87	0.33	0.91	0.27	−0.96	0.17	−0.61	0.59	0.85	0.35

Note: Pearson’s correlation was used to calculate *R* and *p*-values. *p* > 0.05, not significant; *p* ≤ 0.05, significant.

A/C, albumin creatinine ratio; Arg, arginine; Cit, citrulline; eGFR, estimated glomerular filtration rate; HbA1c, haemoglobin A1c; Ile, isoleucine; Orn, ornithine; Phe, phenylalanine; *R*, correlation coefficient; SCr, serum creatinine; Tyr, tyrosine; UCr, urinary creatinine.

The citrulline/ornithine ratios (*p* = 0.01), as well as the concentrations of citrulline (*p* < 0.001) and isoleucine (*p* = 0.04), were significantly higher in the diabetic group compared to the non-diabetic group in stage 1 of CKD (Table 4). Similarly, the ornithine/arginine ratios (*p* = 0.02) and citrulline concentrations (*p* = 0.03) were significantly higher in the diabetic group compared to the non-diabetic group in stage 3. In stage 4, the tyrosine/phenylalanine ratios were significantly (*p* = 0.03) higher, while phenylalanine concentrations were lower in the diabetic group compared to the non-diabetic group.

**TABLE 4:** Differences in amino acid concentrations and clinical parameters between diabetic and non-diabetic patients with declining estimated GFR, Johannesburg, South Africa, November 2019 to January 2021.

Target analyte	Overall			(mL/min per 1.73 m^2^)							
	Diabetic patients	Non-diabetic patients	*p*	15–29		30–59		60–89		≥ 90	
				Diabetic patients	*p*	Diabetic patients	*p*	Diabetic patients	*p*	Diabetic patients	*p*
Arg (mg/L)	13.2 ± 1.20	11.40 ± 13.00	0.19	13.20 ± 4.90	0.40	10.30 ± 2.70	0.33	11.20 ± 1.30	0.47	15.00 ± 1.90	0.08
Cit (mg/L)	13.9 ± 1.30	8.5 ± 1.00	< 0.001	1.49 ± 1.90	0.09	11.20 ± 1.50	0.03	8.80 ± 0.90	0.29	16.90 ± 2.10	< 0.001
Ile (mg/L)	5.00 ± 0.40	4.10 ± 0.60	0.11	3.90 ± 0.30	0.45	4.20 ± 1.30	0.45	4.20 ± 0.40	0.34	5.60 ± 0.50	0.04
Orn (mg/L)	9.00 ± 0.60	7.60 ± 1.00	0.26	8.30 ± 1.30	0.46	8.10 ± 1.60	0.50	8.00 ± 0.80	0.48	9.90 ± 0.90	0.12
Phe (mg/L)	9.90 ± 0.70	8.80 ± 1.60	0.23	5.20 ± 0.90	0.03	6.70 ± 2.20	0.22	8.90 ± 1.30	0.35	11.90 ± 1.00	0.06
Tyr (mg/L)	15.1 ± 1.20	13.20 ± 2.60	0.46	12.3 ± 6.10	0.37	11.50 ± 2.40	0.21	14.10 ± 2.20	0.37	17.00 ± 1.80	0.24
Cit/Orn	0.73 ± 0.10	0.38 ± 0.15	0.01	1.08 ± 0.73	0.19	0.75 ± 0.26	0.07	0.45 ± 0.10	0.18	0.88 ± 0.18	0.01
Arg/Cit	1.31 ± 0.13	1.73 ± 0.37	0.08	0.88 ± 0.88	0.24	1.13 ± 0.18	0.05	1.45 ± 0.20	0.11	1.30 ± 0.19	0.09
Orn/Arg	0.71 ± 0.06	0.49 ± 0.10	0.03	0.84 ± 0.22	0.12	0.90 ± 0.17	0.02	0.69 ± 0.11	0.10	0.67 ± 0.07	0.08
Tyr/Phe	2.94 ± 0.42	1.94 ± 0.48	0.09	4.80 ± 3.94	0.03	4.68 ± 1.87	0.10	3.14 ± 0.70	0.08	2.19 ± 0.34	0.43
HbA1c (%)	9.49 ± 0.21	5.76 ± 0.10	< 0.001	9.27 ± 0.97	0.01	9.47 ± 0.59	< 0.001	9.05 ± 0.34	< 0.001	9.80 ± 0.30	< 0.001
A/C (mg/mmol)	61.80 ± 26.59	4.99 ± 1.77	0.02	515.89 ± 418.89	0.13	89.40 ± 70.00	0.13	23.64 ± 6.15	0.002	27.42 ± 10.73	0.03
ALB (mg/L)	143.65 ± 56.91	21.47 ± 5.62	0.02	188.00 ± 53.35	0.01	583.40 ± 454.65	0.12	83.69 ± 16.59	0.002	68.26 ± 12.41	0.001
UCr (mmol/L)	32.24 ± 5.43	22.41 ± 5.28	0.16	34.51 ± 13.95	0.25	37.94 ± 19.63	0.25	43.38 ± 12.94	0.09	22.78 ± 4.23	0.45
SCr (mg/dL)	1.01 ± 0.05	0.99 ± 0.06	0.03	2.67 ± 0.21	< 0.001	1.46 ± 0.08	< 0.001	0.98 ± 0.02	0.04	0.74 ± 0.02	0.001
Age (years)	56.91 ± 1.32	55.82 ± 3.48	0.26	66.71 ± 5.60	0.05	61.87 ± 3.31	0.08	65.88 ± 1.27	0.004	48.51 ± 1.80	0.09
eGFR (mL/min per 1.73 m^2^)	94.11 ± 4.59	86.94 ± 5.63	0.42	25.29 ± 1.22	< 0.001	48.57 ± 1.93	< 0.001	74.15 ± 1.22	0.001	126.23 ± 7.03	< 0.001

Note: *p* > 0.05, not significant; *p* ≤ 0.05, significant.

A/C, albumin-creatinine ratio; Arg, arginine; Cit, citrulline; HbA1c, haemoglobin A1c; Ile, isoleucine; Orn, ornithine;Phe, phenylalanine; *R*, correlation coefficient; SCr, serum creatinine; Tyr, tyrosine; UCr, urinary creatinine.

## Discussion

The quantitative method developed for the analysis of amino acids in urine was validated and found to be reliable, selective, sensitive, and accurate. We could relate the alterations in urinary amino acid ratios at different stages of CKD to the eGFR and some clinical characteristics associated with the disease. The analysis showed that the amino acid ratios of ornithine/arginine, tyrosine/phenylalanine and arginine/citrulline are affected by the progression of diabetes and can be correlated to renal function. There were significant changes in amino acid ratios between the diabetic and non-diabetic patients in stage 1 of CKD, and this could be utilised to predict the onset of CKD.

The accuracy of the analytical method used to obtain the LLOQ was determined to be within the acceptable range for all the amino acids analysed. The linearity (*R*^2^) ranged from 0.983 to 0.999, the percentage recovery ranged from 90% to 130%, and the precision (%RSD) was less than 30%, except for ornithine. The method was also selective and sensitive as shown by the lowest limit of detection and LLOQ, and all the analytes of interest were well separated in the liquid chromatography and accurately identified by the mass spectrometer. Hence, the method is reliable and can be used to quantify the amino acids of interest within the analytical range of 0.01 mg/L to 5 mg/L.

In diabetics patients, the concentrations of citrulline, isoleucine, phenylalanine and ornithine correlated positively with HbA1c ([*R* = 0.76; *p* = 0.24)], [*R* = 0.83; *p* = 0.17], [*R* = 0.54; *p* = 0.46], [*R* = 0.86; *p* = 0.41]) and eGFR ([*R* = 0.33; *p* = 0.67], [*R* = 0.94, *p* = 0.06], [*R* = 1.00; *p* = 0.004], [*R* = 0.83; *p* = 0.17]). The concentrations of these amino acids decreased with eGFR decline, meaning that the disease and renal function may alter these amino acids. In non-diabetic patients, arginine, citrulline and isoleucine correlated positively with HbA1c, meaning that these amino acid concentrations fluctuate with glucose levels. Even though citrulline showed a positive correlation with HbA1c in both the diabetic and non-diabetic groups, its concentrations levels were significantly (*p* < 0.001) higher in the diabetic group.

A study conducted in France in 2014 reported that citrulline concentration and citrulline/ornithine ratio increased while the tyrosine/phenylalanine ratio decreased (in plasma samples) in the late stages of CKD.^17^ Similarly, in this study, we observed (in urine samples) an increase in citrulline concentrations and citrulline/ornithine ratios in stage 1; however, tyrosine/phenylalanine ratios increased from stage 1 to stage 4 of CKD with decreasing concentrations of phenylalanine. Amino acid concentrations have been reported to decrease in diabetic groups, and this decrease is attributed to the conversion of these amino acids to glucose.^18^ Our findings show that phenylalanine follows this trend, as its concentrations decreased from stage 1 to stage 4 of CKD.

The observed trend can be explained by looking at the metabolic pathways of the studied amino acids that occur in the liver and kidneys. Arginine is converted to ornithine, ornithine to citrulline and citrulline to arginine, while phenylalanine is converted to tyrosine.^17^ Thus, the declining kidney function may affect the proper conversion of citrulline to arginine, as shown also by the significant (*p* = 0.05) decrease in the ratio of arginine/citrulline in stage 3 of CKD. Citrulline is a non-essential amino acid that is mainly synthesised in the gut and then taken up by the kidneys, wherein it is converted to arginine.^19^ The increased concentration of citrulline in diabetic patients could be attributed to the inability of the kidneys to carry out this function.

The kidneys convert 50% of the total body phenylalanine to tyrosine through hydroxylation by the action of the phenylalanine-4-hydroxylase enzyme.^19^ At stage 4 of CKD, the tyrosine/phenylalanine ratios were significantly different between the diabetic and non-diabetic groups, and the phenylalanine concentrations were lower in the diabetic group. The metabolism of phenylalanine has two outcomes – the production of tyrosine, and the complete oxidation of phenylalanine to carbon dioxide and water to drive the production of glucose.^20^ We observed an increase in tyrosine/phenylalanine ratios with eGFR decline, which suggests an increased production of tyrosine in the kidneys as renal function decreases and could explain the low phenylalanine concentrations in stage 4 of CKD.

### Limitations

The exact cut-off values for amino acid concentrations and ratios, which indicate the onset of diabetic nephropathy or CKD, could not be determined. Also, the sample size for non-diabetic patients was below 30 (*n* = 22), which is the acceptable size for accurate statistical evaluation, and this may have influenced the results of comparisons between the two groups of patients. A more detailed statistical evaluation would have to be conducted to indicate early CKD using amino acid ratios.

### Conclusion

The amino acid concentrations and ratios in human urine samples could provide valuable information about the onset and progression of diabetic nephropathy as there appeared to be significant correlations between the amino acid concentrations and declining eGFR. Amino acid ratios (ornithine/arginine and tyrosine/phenylalanine) are affected by decreased renal function, and citrulline/ornithine ratios may predict early CKD.
